# Comparative analysis of the tear protein profile in herpes simplex virus type 1 epithelial keratitis

**DOI:** 10.1186/s12886-020-01626-3

**Published:** 2020-08-31

**Authors:** Hua Yang, Xiaozhao Yang, Yani Wang, Xuan Zheng, Yi Zhang, Yan Shao

**Affiliations:** 1 Department of Ophthalmology, Xi’an No.1 Hospital, Xi’an, 710002 Shaanxi Province China; 2 Shaanxi Institute of Ophthalmology, Xi’an, 710002 Shaanxi Province China; 3 Shaanxi Key Laboratory of Ophthalmology, Xi’an, 710002 Shaanxi Province China; 4 Clinical Research Center for Ophthalmology Diseases of Shaanxi Province, Xi’an, 710002 Shaanxi Province China; 5 First Affiliated Hospital of Northwestern University, Xi’an, 710002 Shaanxi Province China

**Keywords:** HSV-1 epithelial keratitis, Tears, Proteome, Mass spectrometry, Bioinformation, ELISA

## Abstract

**Background:**

Herpes simplex virus type 1 (HSV-1) keratitis is a major cause of corneal blindness in the world, and an in-depth understanding of its pathogenesis may help improve existing diagnosis and treatment. The purpose of this study is to compare and analysis the total tear protein profile of HSV-1 epithelial keratitis patients, and to quantify the potential candidate biomarkers of HSV-1 epithelial keratitis.

**Methods:**

We investigated the proteome in tear fluid from three HSV-1 epithelial keratitis patients and three healthy control subjects using nano-scale liquid chromatography-tandem mass spectrometry (nLC-MS/MS) analysis. Functional annotation of differentially expressed proteins was done with the Gene Ontology (GO) analysis. ELISA was done to quantify the potential candidate biomarkers in 26 clinical cases.

**Results:**

Tear fluid from three HSV-1 epithelial keratitis patients and three healthy control subjects contained a total of 1275 proteins and 326 proteins were unique to tear fluid of HSV-1 epithelial keratitis patients. Bioinformatics analysis revealed that tear proteins from HSV-1 epithelial keratitis patients may be involved in metabolic processes, antigen presentation, inflammatory response, and in the TNF-mediated and T cell receptor pathways. Furthermore, IL1A, IL12B, DEFB4A, and CAMP, which are associated with the inflammatory response and inhibition of viral infection, were significantly more abundant in the HSV-1 epithelial keratitis patients than in the healthy control subjects.

**Conclusions:**

This study reports the proteomic profile of tears in HSV-1 epithelial keratitis for the first time and identifies a number of unique differentially expressed proteins.

## Background

Herpes simplex virus type 1 (HSV-1) is the most important pathogen causing ocular diseases in the world [1]. More than 40,000 new cases caused by HSV-1 infection each year, which range from mild corneal epithelial inflammation to severe chronic corneal ulcerations [2, 3]. Despite the diagnosis and treatment of eye disease have enormous progress, HSV-1 keratitis still remains a leading cause of visual impairment and eventually blindness, and there is currently no effective vaccine available [4, 5]. Corneal disease due to HSV-1 commonly presents as epithelial keratitis which, though usually self-limiting, may persist or progress without treatment [2]. The use of antiviral drugs can effectively alleviate the HSV-1 epithelial keratitis process; however, their toxicity is often a problem, and efficacy can be limited due to the development of drug-resistant mutants [6]. The increasing prevalence of acyclovir- and multidrug-resistant HSV-strains is the main reason for treatment failure of inflammatory corneal HSV-infections [7]. Therefore, studying the pathogenesis of HSV-1 epithelial keratitis is essential for ensuring its early diagnosis and developing novel, well-tolerated treatment options for patients.

Tears are a complex biological mixture known to have electrolytes, lipids, enzymes, metabolites, small organic molecules, and proteins [8]. Their main function is to provide lubrication and nutrition to the cornea, and become the first line of defense against pathogens [8]. Proteins are the most abundant components in human tears, accounting about 95% of their dry weight. Therefore, changes in tear protein composition can directly reflect the physiological and pathological state of the eye tissue [9]. High-throughput proteomics technology provides a comprehensive analysis of the tear proteome and can be used as a screening method for ocular diseases such as dry eye disease [10, 11], bacterial keratitis [12], fungal keratitis [13, 14], diabetic retinopathy [15, 16], and thyroid-associated eye disease [17]; systemic diseases like breast cancer [18, 19] and neurological disorders [20]; and for discovering potential new therapeutic targets for these clinical situations. Therefore, studying the characterization of proteomic changes associated with HSV-1 epithelial keratitis will help to elucidate its pathogenesis, and further identify the therapeutic targets of disease.

In this study, we aimed to investigate changes in the tear proteome in HSV-1 epithelial keratitis patients. Using nano-scale liquid chromatography-tandem mass spectrometry (nLC-MS/MS), we generated tear proteome data of three HSV-1 epithelial keratitis patients, performed Gene Ontology (GO) analysis, and analyzed the levels of four specific proteins (IL1A, IL12B, DEFB4A, and CAMP) identified in tears of 26 HSV-1 epithelial keratitis patients. In summary, we identified novel proteins and pathways associated with the molecular mechanisms of HSV-1 epithelial keratitis. This study is the first report of tear proteome data from HSV-1 epithelial keratitis patients.

## Methods

### Patients and tear collection

This study conformed to the ethical guidelines of Declaration of Helsinki, and was performed according to the institutional ethics committee of the Xi’an No.1 Hospital. Twenty-six HSV-1 epithelial keratitis patients who first visit the clinic (including 14 males and 12 females, with a mean age of 35.60 ± 14.78 years) were recruited from our hospital. Twenty-six healthy control subjects (including 13 males and 13 females, with a mean age of 32.52 ± 15.43 years) were recruited from the community, and they had no prior ocular complaints, no history of eye medication and wearing contact lenses, and were not on any systemic medication. There was no significant difference in gender and age between the two groups (*P* > 0.05). All patients and healthy control subjects were examined by slit-lamp biomicroscopy. In addition, corneal swabs or tears were taken aseptically and HSV-1 was identified as the causative virus by real-time PCR analysis and rapid culture [21]. After informed consent, reflex tear samples (100 μl per sample) were collected from the inferior temporal tear meniscus using calibrated 10-μl glass microcapillary tubes (Blaubrand intraMark, Wertheim, Germany). After collection, tear samples were centrifuged at 10,000 g for 10 min to remove cellular debris, and stored at − 80 °C.

### Reduction, alkylation and digestion

To digest the tear proteins, 10 μg samples were treated with denaturing reagent RapiGest SF according to the instructions (Waters, Manchester, UK). Then, the samples were reduced with 10 mM dithiothreitol (Sigma-Aldrich) at 37 °C for 30 min, alkylated with 25 mM iodoacetamide (Sigma-Aldrich) at 25 °C for 15 min in the dark, and digested with 0.2 μg trypsin (Promega) at 37 °C for 12 h. Finally, the digestion reaction was stopped by addition of 1 μL 1% formic acid (Sigma-Aldrich).

### nLC-MS/MS analysis

Tear samples from 3 HSV-1 epithelial keratitis patients and 3 healthy control subjects were analyzed using a nanoflow high pressure liquid chromatography system interfaced to an LTQ-Orbitrap XL mass spectrometer (Thermo Fisher Scientific, Germany). Briefly, 1 μg sample was loaded onto a 180 μm × 20 mm (ID) C18 column precolumn (5 μm, 200 Å, Waters Corporation) and separated on a commercial 75 μm × 150 mm C18 column (5 μm, 100 Å, Nikkyo Technology). The analytical separation was run for 85 min, details as follows: starting from 5 to 35% of solvent B for 55 min, from 35 to 80% of solvent B for 10 min, holding at 80% of solvent B for 2 min, and equilibrating the column at 5% of B for 18 min (solvent B: 0.1% formic acid in acetonitrile). Key parameter settings for the LTQ Orbitrap XL mass spectrometer were as follows the manufacturer’s specifications. The resulting spectra were recorded for each run and searched using Mascot version 2.2.03, and parameters were as follows: one missed cleavage, the fixed modification was carbamidomethylation of cysteines, the variable modification was oxidation of methionine, peptide mass tolerance was 10 ppm, fragment mass tolerance was 0.6 Da, and false discovery rate was less than 1%.

### Bioinformatics analysis

A list of the proteins’ Gene IDs was submitted to DAVID 6.8 for GO enrichment analysis (http://david.abcc.ncifcrf.gov/home.jsp). Each cluster is given a “Fold enrichment” score based on the Fisher Exact Statistics, and *P* values < 0.05 were considered as significant. The unique protein spectra of tears of HSV-1 epithelial keratitis patients were subjected to hierarchical clustering with Cluster 3.0 software (http://bonsai.hgc.jp/~mdehoon/software/cluster/software.htm) and visualized with the TreeView software (http://sourceforge.net/projects/jtreeview/files/).

### Enzyme-linked immunosorbent assay (ELISA)

Tears samples collected from 26 HSV-1 epithelial keratitis patients and 26 healthy control subjects (including samples in MS experiment) were analyzed by ELISA using the Human IL-1 alpha/IL-1F1 and IL-12 p70 Quantikine ELISA Kits (R&D Systems, Minneapolis, MN, USA), and Human DEFB4A and CAMP ELISA Kits (MyBioSource, San Diego, CA, USA). Absorbance was measured at 450 nm using a microplate reader.

### Statistical analysis

Data are expressed as the mean ± standard error of the mean. Sample comparisons were performed using the Student’s t-test for independent samples. Statistical analysis was conducted with SPSS version 16.0. (SPSS Inc., Chicago, IL, USA). *P*-values < 0.05 were considered to indicate a statistically significant difference.

## Results

### Proteomes of tears from HSV-1 epithelial keratitis patients

**Fig. 1:**
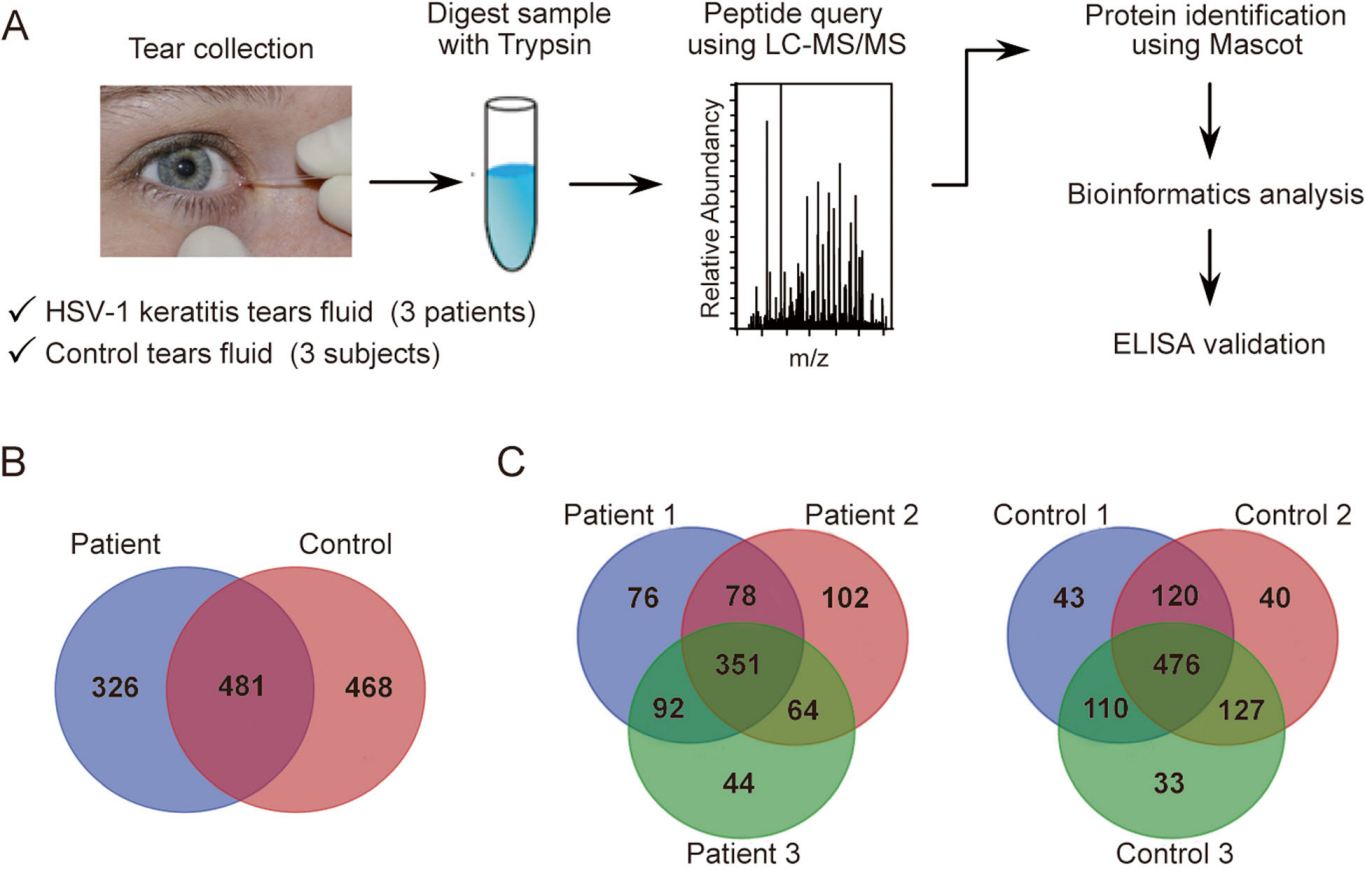
Schematic diagram of proteomics experiments and Venn diagrams of the identified proteins. **a** Schematic overview of the proteomic analysis of tear fluid. **b** Tear proteins unique to and shared between 3 HSV-1 epithelial keratitis patients and 3 healthy control subjects. **c** Unique and shared tear proteins of HSV-1 epithelial keratitis patients (left panel) and healthy control subjects (right panel)

We investigated the protein composition of tears from HSV-1 epithelial keratitis patients (*n* = 3) and healthy control subjects (n = 3) according to the schematic workflow shown in Fig. 1a. In total, 807 and 950 proteins were identified in the HSV-1 epithelial keratitis patients (S1 Table) and healthy control subjects (S2 Table), respectively. Keratins have been excluded from our data, because they are well-known as common laboratory contaminants in proteomic analyses. Among the identified proteins in HSV-1 epithelial keratitis patients and healthy control subjects, only 481 were common to both groups, and 326 proteins were found to be unique to HSV-1 epithelial keratitis patients (Fig. 1b, and Table S3). As expected, highly abundant proteins such as lactotransferrin, lipocalin 1, lipocalin 2, albumin, lysozyme, complement C3, and immunoglobulin heavy constant alpha 1 were present in tears of individuals from both groups. Most of these proteins are involved in inflammatory and antimicrobial processes, and play an important role in maintaining a clean ocular environment [22]. In the three HSV-1 epithelial keratitis patients, 597, 595, and 551 proteins were identified, respectively, while 351 (43.5%) proteins were identified in all three patients (Fig. 1c). In the three healthy control subjects, 749, 763, and 746 proteins were identified, respectively, while 476 (50.2%) proteins were identified in all three subjects (Fig. 1c).

### Functional analysis of unique proteins in tears of HSV-1 epithelial keratitis patients

**Fig. 2:**
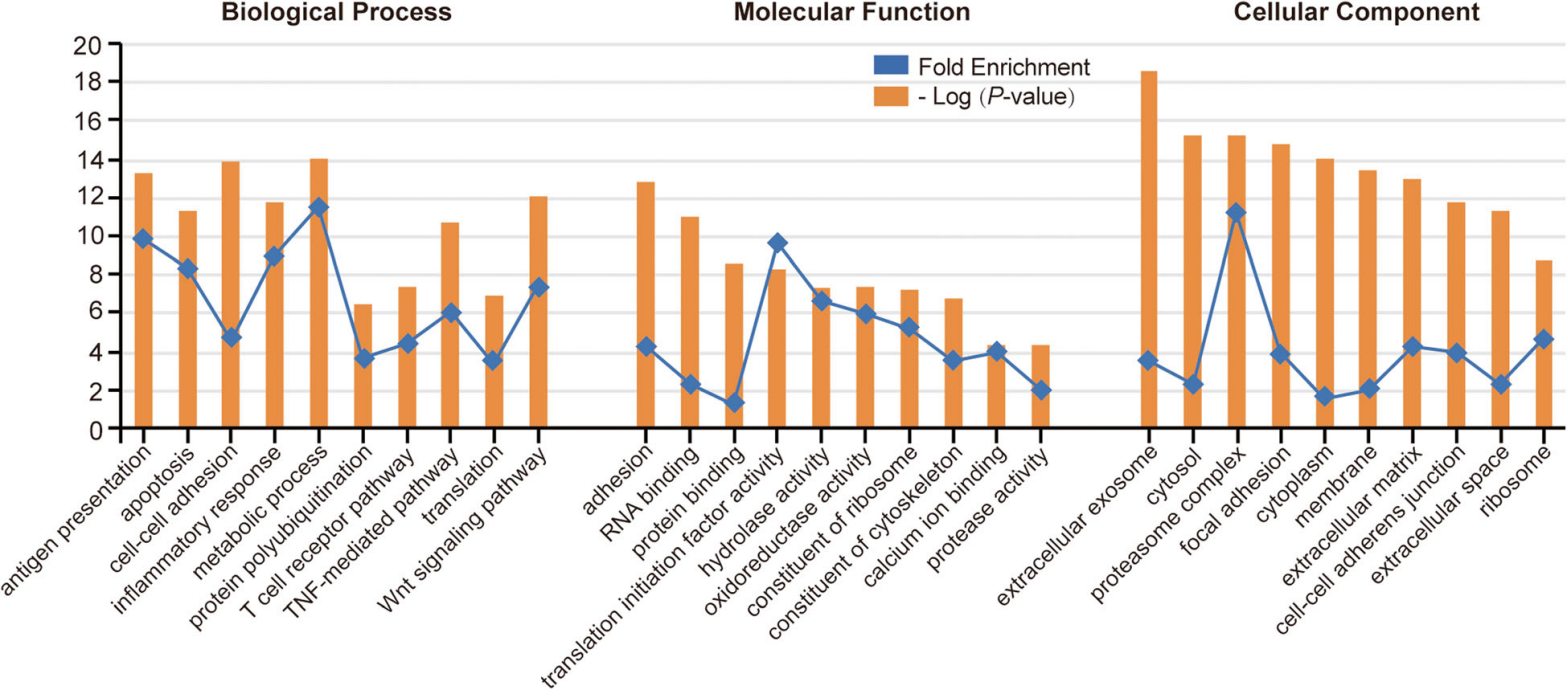
Functional analysis of unique proteins in tears of HSV-1 epithelial keratitis patients. The list of the 326 proteins’ Gene IDs was submitted to DAVID enrichment analysis tool, and the top 10 statistically significant categories are listed in biological processes, molecular functions and cellular components. The *P*-value less than 0.05 indicates the significance level of the enrichment. To show *P*-value in this graph together with fold enrichment, negative logarithmic *P*-value was plotted

Based the DAVID enrichment analysis tool, the 326 proteins unique to HSV-1 epithelial keratitis patients were classified according to their biological processes, molecular functions and cellular components (Fig. 2). The top categories for biological processes were metabolic process, antigen presentation, inflammatory response, and TNF-mediated and T cell receptor pathways. This was in line with previous virus- and cell-based studies showing that HSV-1 infection induces the innate immune response, and upregulates inflammatory factors for inhibiting viral infection [23]. An unexpectedly large number of proteins were involved in cell-cell adhesion (such as CD81), and translation (such as EIF4E). This may indicate that HSV-1 interacts with corneal epithelial cells during infection, and may support the idea of adhesion, transcription, and translation of viruses [24, 25]. In addition, some proteins were assigned to the categories apoptosis, the Wnt signaling pathway, and protein polyubiquitination. We suggested that these proteins may be involved in events involving the corneal epithelial cell damage after viral infection [26]. The assignment of molecular functions revealed that HSV-1 epithelial keratitis tear proteins, preferentially bind to proteins associated with translation initiation factor activity. The other large ontology group of the tear’s interactome was enzyme activity, which was mainly linked to hydrolase, oxidoreductase, and protease activity. Enrichment of these enzymes may be associated with corneal cell damage or inhibition of viral infection [27, 28]. Proteins were also categorized according to cellular components. We found that although a large number of proteins were assigned to the categories extracellular matrix and extracellular exosome system, proteins were highly enriched for the proteasome complex and cytosol. This suggests that tear protein activity in HSV-1 infections is a dynamic process, involving the communication between multiple organelles.

### Cluster analysis of the 326 tear proteins unique to HSV-1 epithelial keratitis patients

**Fig. 3:**
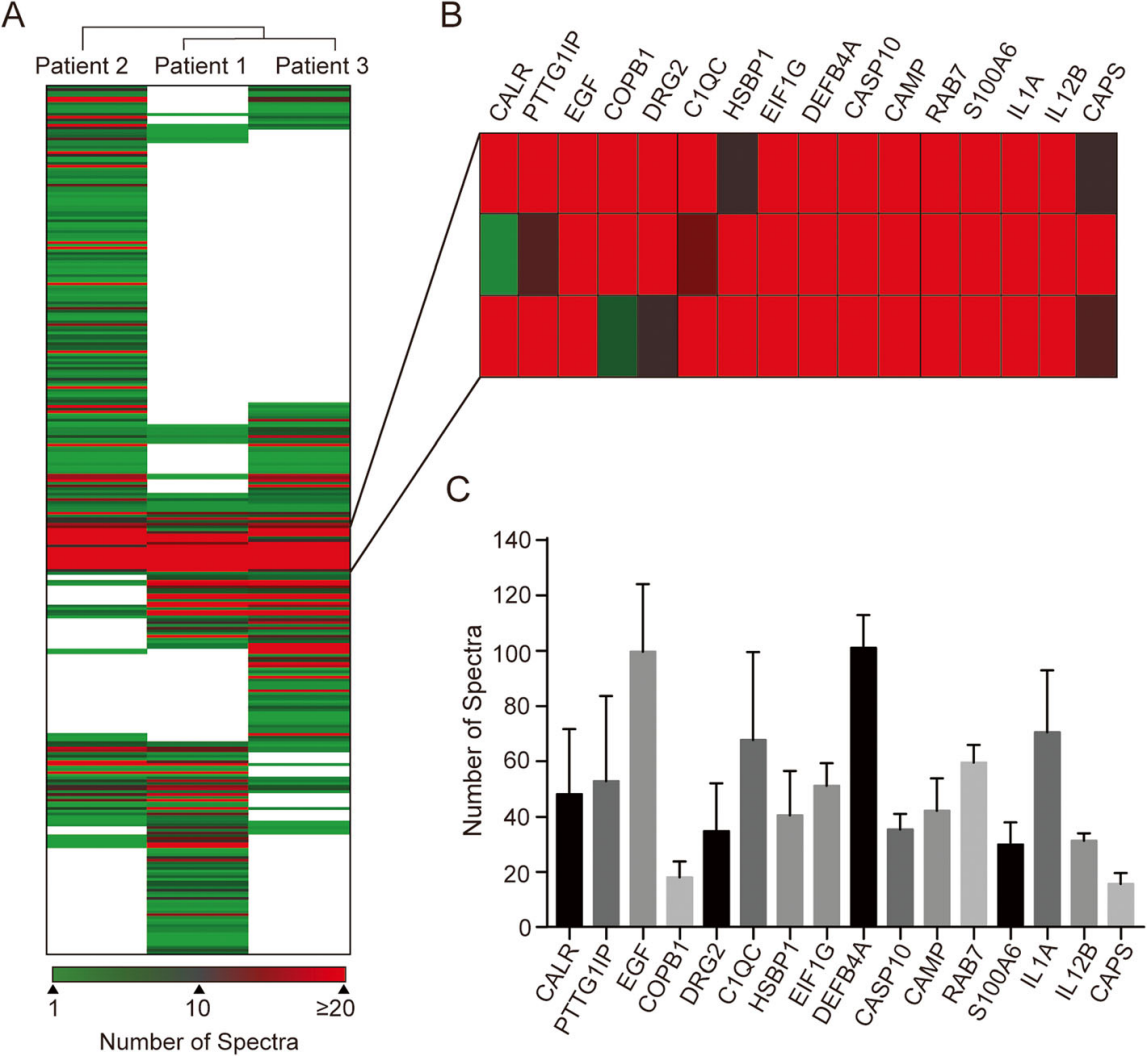
Cluster analysis of the 326 unique tear proteins to the three HSV-1 epithelial keratitis patients. **a** Hierarchical two-dimensional clustering of the proteins based on spectral counting. Each row represents an individual protein and each column an individual patient. **b** Shown are highly abundant proteins present in the tears of all three patients. **c** Spectral counting analysis of the highly abundant proteins in three HSV-1 epithelial keratitis patients

To further investigate the proteins that may distinguish between HSV-1 epithelial keratitis patients and healthy controls, we analyzed the protein expression profiles of the 326 unique proteins of HSV-1 epithelial keratitis patients. We then ran a hierarchical cluster analysis of these proteins based on the number of protein spectra and cluster data were assembled to provide a heat map (Fig. 3a). Each row represents an individual protein signal and each column an individual sample. The dendrogram above the heat map shows the similarity in protein expression profiles of the samples. By comparing the differences in the tear proteomes of the three HSV-1 epithelial keratitis patients, it is clear that the tear proteome comprises proteins unique to each patient and proteins common to the three patients. Surprisingly, although several proteins including IL6, S100A8, and VEGFA were only detected in one or two patients, previous studies reported their involvement in HSV-1 epithelial keratitis [29–31]. Proteins common to all three patients included factors considered to be involved in inhibition of viral infection (such as C1QC, CAMP, DEFB4A, and S100A6), proteins associated with the inflammatory response (IL1Α and IL12B) and cell damage (CASP10, EGF, HSBP1, and DRG2), and proteins with an unknown function in keratitis (PTTG1IP, CALR, CAPS, COPB1, and RAB7A) (Fig. 3b). To select appropriate proteins for ELISA analysis, we analyzed the abundance of these proteins for the three HSV-1 epithelial keratitis patients (Fig. 3c).

### Quantification analysis by ELISA

**Fig. 4:**
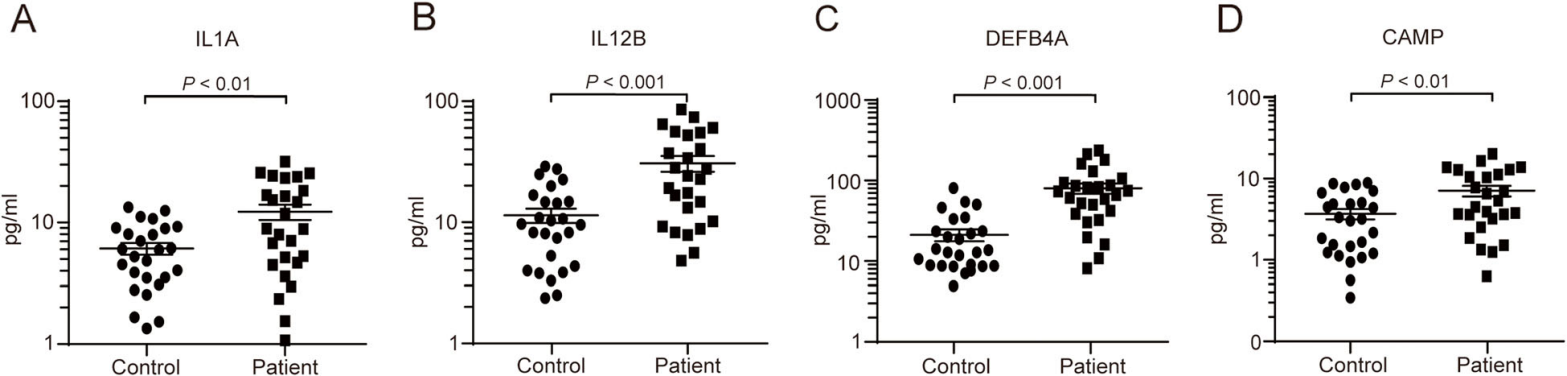
The concentration of IL1Α (**a**), IL12B (**b**), DEFB4A (**c**), and CAMP (**d**) in 26 HSV-1 epithelial keratitis patients and 26 healthy control subjects were measured using ELISA. Data represents mean ± SEM of three independent experiments and were analyzed using an unpaired t-test

To test whether these protein candidates would be detectable in clinical samples, we selected four proteins (IL1Α, IL12B, DEFB4A, and CAMP) for ELISA analysis based on the following criteria: abundance, repeatability, and association with viral infection. Compared with their concentrations in 26 healthy control subjects, concentrations of IL1Α, IL12B, DEFB4A, and CAMP were significantly higher in 26 HSV-1 epithelial keratitis patients. These results show that our mass spectrometry data was accurate and reliable. While the mean concentrations of IL1Α, IL12B, DEFB4A, and CAMP in healthy control subjects were (6.10 ± 0.68 pg/ml), (10.78 ± 1.45 pg/ml), (21.24 ± 3.50 pg/ml) and (3.82 ± 0.57 pg/ml) respectively, mean concentrations of IL1Α, IL12B, DEFB4A, and CAMP in HSV-1 epithelial keratitis patients were (12.26 ± 1.78 pg/ml) (*P* < 0.01, Fig. 4a), (32.28 ± 4.81 pg/ml) (*P* < 0.001, Fig. 4b), (77.71 ± 11.13 pg/ml) (*P* < 0.001, Fig. 4c), and (7.34 ± 1.11 pg/ml) (*P* < 0.05, Fig. 4d), respectively.

## Discussion

Protein profiles of tears from HSV-1 infected individuals may show increased levels of proinflammatory cytokines, alterations in signaling molecules and hormone levels, presence of autoantibodies, factors involved in apoptosis, and many other changes [32]. Therefore, alterations in tear protein profiles are indicative of the processes underlying HSV-1 epithelial keratitis, and identification of marker proteins can provide information on the disease severity, as well as on the underlying pathology.

In this study, we identified 1275 proteins in the tear from three HSV-1 epithelial keratitis patients and three healthy control subjects using nLC-MS/MS. *Zhou* et al has reported a high-confidence human tear proteome reference set which contains 1543 proteins (from four healthy subiects) [33]. By comparing our tear proteome data with their tear proteome data reference set, we found that 1026 proteins (80.5% of total 1275 proteins) were common, while 952 proteins were identified in healthy control subjects, and 596 proteins were identified in HSV-1 epithelial keratitis patients. By comparing the differences in the tear proteomes between HSV-1 epithelial keratitis patients and healthy control subjects, 326 proteins were found to be unique to HSV-1 epithelial keratitis patients. This result suggest that infection of the cornea with HSV-1 leads to significant change in the protein composition of tears, which may be due to the abnormal secretion of lacrimal glands and corneal cell damage [9]. GO enrichment analysis suggest that these unique proteins do not only participate in the host immune response, but also in cell damage and stress response. Through cluster analysis, we obtained several proteins with highly abundance and repetition rate in the tear from three HSV-1 epithelial keratitis patients (Fig. 3b-c). Most of these proteins are related to inhibition of viral infection, inflammatory response and cell damage, and may serve as a target for studying the pathogenesis and treatment of HSV-1 epithelial keratitis. It has to be noted that although some proteins (IL-6, S100A8 and VEGFA) were present in low abundance or in one or two patients only, they also play an important role in the process of HSV-1 keratitis [29–31].

HSV-1 epithelial keratitis is thought to be caused by the virus replicating in and destroying epithelial cells [2]. The lesions start as punctuate vesicular eruptions in the corneal epithelium, progressing to a stellate erosion, but quickly coalesce into dendritic shaped lesions [34]. In order to suppress the infection and replication of the virus, the expression of various inflammatory factors and antiviral proteins were increased in corneal epithelial cells [35]. Our mass spectrometry data showed the spectrum of several inflammatory factors were increased in tears of HSV-1 epithelial keratitis patients, such as IL1A and IL12B (Fig. 3 and S1 Table). ELISA also confirmed that IL1A and IL12B were significantly elevated in patients with HSV-1 epithelial keratitis (Fig. 4). The current research shows that IL1A and IL-12B are important proinflammatory cytokines in HSV-1 epithelial keratitis and are not only produced by activated macrophages and lymphocytes, but also by corneal epithelium and keratocytes [36]. When corneal epithelial cells are damaged by virus invasion, IL1 and IL12B are released as early warning signal [37]. Local release of IL1A and/or IL12B triggers a series of events including an increase in the activity of inflammatory mediators such as prostaglandins, leukotrienes, and nitric oxide [38, 39]. At the same time, upregulated inflammatory mediators promote the accumulation of neutrophils, macrophages, Langerhans cells, and lymphocytes [38, 39]. In addition to increased expression of inflammatory factors, related antiviral proteins were also increased in tears of HSV-1 epithelial keratitis patients. Human DEFB4A (also named β-defensin-2), an inducible, anti-microbial peptide with a molecular mass of 4–6 kD, acts as an endogenous antibiotic in the defense against microbial infections [40]. DEFB4A is only found occasionally in healthy ocular surface tissue, but it is found more frequently in infected/inflamed ocular surface tissue [41]. CAMP (also named LL-37), the sole know member of the cathelicidin family of peptide expressed in humans, is a multifunctional host defense molecule essential for normal immune responses to infection and tissue injury [42]. Studies have shown that corneal and conjunctival epithelia express CAMP as part of mucosal innate immunity to protect against bacterial and viral ocular infections [43]. Notably, in a rabbit model of corneal injury, epithelial expression of CAMP was upregulated and levels of CAMP in the tears correlated with wound closure [44]. Such observations have led to the suggestion that DEFB4A and CAMP may be important for ocular surface wound healing.

This study is the first report of tear proteome data from HSV-1 epithelial keratitis, which may deepen our understanding of molecular mechanisms behind HSV-1 epithelial keratitis. However, there are some possible limitations in this study, including the small sample size, the lack of clinical data related to disease severity. In addition, due to the limited volume of tear fluid, the collection technique will affect the protein profile of the tear sample. Future, we will focus on the correlation between the unique proteins of HSV-1 epithelial keratitis and the severity and/or prognosis of the disease. This requires a larger sample size and follow-up of patients to assess the clinical significance of these markers.

## Conclusions

The present study provides an overview of the tear protein profile changes in HSV-1 epithelial keratitis patients through a proteomic approach, and identified 326 unique proteins which may have a bearing on pathogenesis and disease progression. This unique tear protein list may be used as a reference list for tear biomarker search of HSV-1 epithelial keratitis.

## Supplementary information

**Additional file 1: Table S1.** Tear proteome of HSV-1 epithelial keratitis patients.

**Additional file 2: Table S2.** Tear proteome of healthy control subjects.

**Additional file 3: Table S3.** The 326 unique proteins of HSV-1 epithelial keratitis patients.

## Data Availability

The datasets used and/or analysed during the current study are available from the corresponding author on reasonable request.

## Abbreviations

HSV-1: Herpes simplex virus type 1
nLC-MS/MS: Nano-scale liquid chromatography-tandem mass spectrometry
GO: Gene Ontology
CD81: CD81 molecule
EIF4E: Eukaryotic translation initiation factor 4E
C1QC: Complement C1q C chain
CAMP: Cathelicidin antimicrobial peptide
DEFB4A: Defensin beta 4A
S100A6: S100 calcium binding protein A6
IL1A: Interleukin 1 alpha
IL12B: Interleukin 12B
CASP10: Caspase 10
EGF: Epidermal growth factor
HSBP1: Heat shock factor binding protein 1
DRG2: Developmentally regulated GTP binding protein 2
PTTG1IP: Pituitary tumor-transforming 1 interacting protein
CALR: Calreticulin
CAPS: Calcyphosine
COPB1: Coatomer protein complex subunit beta 1
RAB7A: RAB7A, member RAS oncogene family
